# The Role of Cgrp-Receptor Component Protein (Rcp) in Cgrp-Mediated Signal Transduction

**DOI:** 10.1100/tsw.2001.435

**Published:** 2001-10-24

**Authors:** M. A. Prado, B. Evans-Bain, S. L. Santi, I. M. Dickerson

**Affiliations:** Department of Physiology and Biophysics, The University of Miami School of Medicine, Miami, FL, USA

## Abstract

The calcitonin gene-related peptide (CGRP)-receptor component protein (RCP) is a 17-kDa intracellular peripheral membrane protein required for signal transduction at CGRP receptors. To determine the role of RCP in CGRP-mediated signal transduction, RCP was depleted from NIH3T3 cells using antisense strategy. Loss of RCP protein correlated with loss of cAMP production by CGRP in the antisense cells. In contrast, loss of RCP had no effect on CGRP-mediated binding; therefore RCP is not acting as a chaperone for the CGRP receptor. Instead, RCP is a novel signal transduction molecule that couples the CGRP receptor to the cellular signal transduction machinery. RCP thus represents a prototype for a new class of signal transduction proteins that are required for regulation of G protein-coupled receptors.

